# Downregulation of TAP1 in Tumor-Free Tongue Contralateral to Squamous Cell Carcinoma of the Oral Tongue, an Indicator of Better Survival

**DOI:** 10.3390/ijms21176220

**Published:** 2020-08-27

**Authors:** Nima Attaran, Xiaolian Gu, Philip J. Coates, Robin Fåhraeus, Linda Boldrup, Torben Wilms, Lixiao Wang, Nicola Sgaramella, Katarina Zborayova, Karin Nylander

**Affiliations:** 1 Department of Clinical Sciences/ENT, Umeå University, SE-901 85 Umeå, Sweden; attaran.nima1@gmail.com (N.A.); torben.wilms@umu.se (T.W.); Katarina.Zborayova@regionvasterbotten.se (K.Z.); 2 Department of Medical Biosciences, Umeå University, SE-901 85 Umeå, Sweden; xiaolian.gu@umu.se (X.G.); linda.boldrup@umu.se (L.B.); lixiao.wang@umu.se (L.W.); sgaramellanicola12@gmail.com (N.S.); 3 RECAMO, Masaryk Memorial Cancer Institute, Zluty kopec 7, 656 53 Brno, Czech Republic; p.j.coates91259@gmail.com; 4 Institut de Génétique Moléculaire, Université Paris 7, Inserm UMR 1162, 750 13 Paris, France; robin.fahraeus@inserm.fr

**Keywords:** TAP1, SCCOT, field cancerization, MHC I

## Abstract

Oral cancers are surrounded by epithelium that histologically might seem normal, but genetically has aberrations. In patients with squamous cell carcinoma of the oral tongue (SCCOT), it is therefore important to study not only the tumor but also the clinically tumor-free contralateral tongue tissue that remains in the patient after treatment to map changes of prognostic and/or diagnostic value. The transporter associated with antigen processing (TAP) dimer is a key factor in the process of activating cytotoxic T cells. By downregulating the expression of TAP, tumor cells can escape cytotoxic T cell recognition. Biopsies from tumor and clinically tumor-free contralateral tongue tissue in 21 patients with SCCOT were analyzed together with tongue biopsies from 14 healthy individuals, which served as the control group. Dividing patients into *TAP1*-high and *TAP1*-low groups according to the median *TAP1* level in tumor-free samples showed that patients with lower *TAP1* mRNA levels in tumor-free samples had better overall (*p* = 0.003) and disease-free survival (*p* = 0.002). The results showing that *TAP1* levels in tumor-free tongue tissue contralateral to the SCCOT correlate with survival is an important contribution to early diagnosis and follow up of SCCOT.

## 1. Introduction

In Sweden, approximately 160 new cases of squamous cell carcinoma of the tongue (SCCOT) are detected annually, making it the most frequent tumor in the oral cavity. Despite advances in various treatment modalities resulting in improved local control, the long-term survival rates of patients with SCCOT has only marginally improved [1]. More than one-third of these tumors have regional lymph node metastasis already at diagnosis, significantly reducing the survival [2]. There is thus a great need to explore novel biomarkers enabling detection of tumors prior to clinical manifestation. 

In 1953 Slaughter et al. introduced the field cancerization model, showing that oral cancers are surrounded by epithelium that is not unaffected by the cancerous development. When surgically removing an oral cancer, seemingly benign epithelium preconditioned to develop into cancer is accordingly left behind [3]. This could help explain the high incidence of local recurrence and multiple second primary tumors seen in patients with SCCOT and other types of squamous cell carcinoma of the head and neck (SCCHN). Fifty years later, genetically altered fields in the case of intraoral SCC can be detected as far as 7 cm from the tumor, implying that, although clinically normal, the main part of the oral cavity is affected by genetic alterations [4]. Recently, it has been suggested to consider field cancerization as representing an altered microenvironment rather than purely genetic alterations [5]. In the case of SCCOT, it can thus be of value to study not only the tumor itself but also the clinically tumor-free adjacent tongue tissue in order to map changes indicative of a tumor, novel or relapse, before any clinical signs are present.

Our own recent studies of clinically tumor-free tongue tissue contralateral to the SCCOT showed increased cytolytic T cell activity compared to normal tongue from volunteers with no evidence of SCCOT [6]. This cytolytic activity is a measure of cytotoxic proteins released by CD8^+^ T cells and NK cells, important players in the adaptive and innate immune responses, respectively [7]. 

A key factor in the process of cytotoxic T cell activation is the TAP1/TAP2 peptide transporter complex that brings antigen peptide substrates into the endoplasmic reticulum, where they are processed and loaded onto major histocompatibility class I (MHC-I) for export to the cell surface via the Golgi apparatus [8,9,10]. By downregulating the expression of transporter associated with antigen processing, TAP, causing loss of MHC I complexes on the surface, tumor cells can escape cytotoxic T cell recognition [11]. Mutations affecting TAP1 are frequently seen in human tumors and metastatic tumors more often show loss of function of TAP than primary tumors [11,12]. In addition, viruses such as human papilloma virus, HPV, cytomegalovirus, CMV, and Epstein Barr virus, EBV, downregulate TAP1 to avoid immune surveillance [11].

Based on our previous findings of an increased cytolytic activity in tumor-free tongue tissue contralateral to an SCCOT [6] remaining in the patient after treatment, we focused on *TAP* expression in these tissues.

## 2. Results 

### 2.1. Progressive Upregulation of TAP1 from Control to Tumor-Free to Tumor

**Figure 1:**
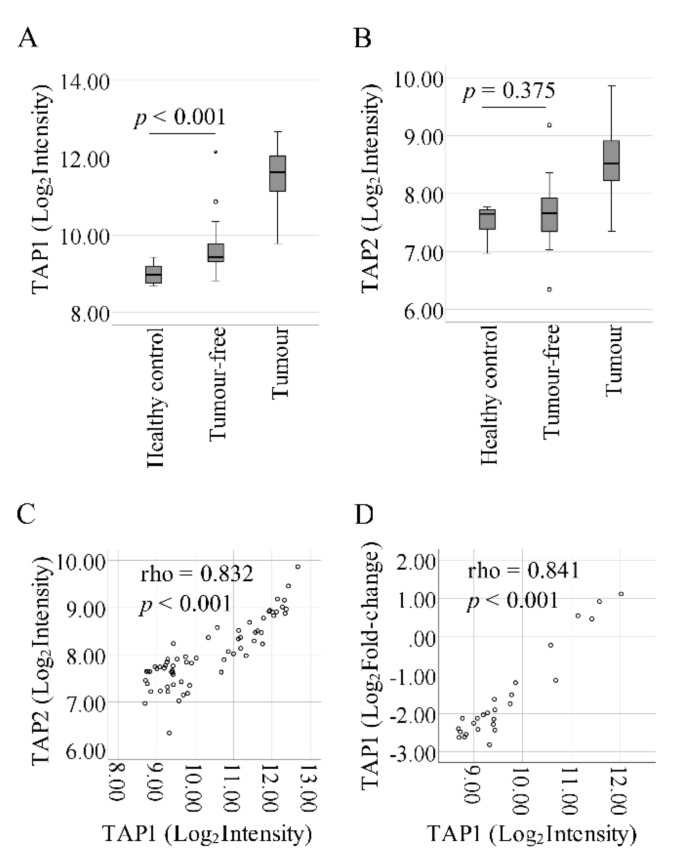
Microarray results of (**A**) *TAP1* and (**B**) *TAP2* mRNA levels in tongue from healthy volunteers, tumor-free samples and SCCOT samples. (**C**) Correlation between *TAP1* and *TAP2* levels (*p* < 0.001). (**D**) Real-time quantitative PCR (RT-qPCR) confirmation of microarray data for *TAP1* (spearman correlation coefficient: 0.841, *p* < 0.001).

Analysis of previously published microarray results [6,13] showed that *TAP1* mRNA is significantly upregulated in tumor-free tongue compared to healthy controls (fold change = 1.65), and further upregulated in tumor samples (Figure 1A). For *TAP2,* there was no significant difference in mRNA between tumor-free and healthy controls (Figure 1B), whereas a significant correlation between *TAP1* and *TAP2* levels was seen (Figure 1C). To confirm the microarray data, RT-qPCR was used for measuring *TAP1* levels in 10 of the healthy tongue controls and 8 paired tumor-free and SCCOT samples. There was a significant correlation between microarray and RT-qPCR results for *TAP1* (spearman correlation coefficient: 0.841, *p* < 0.001; Figure 1D). 

### 2.2. Expression of TAP1 in Tumor-Free Samples Confers Prognostic Information

**Figure 2:**
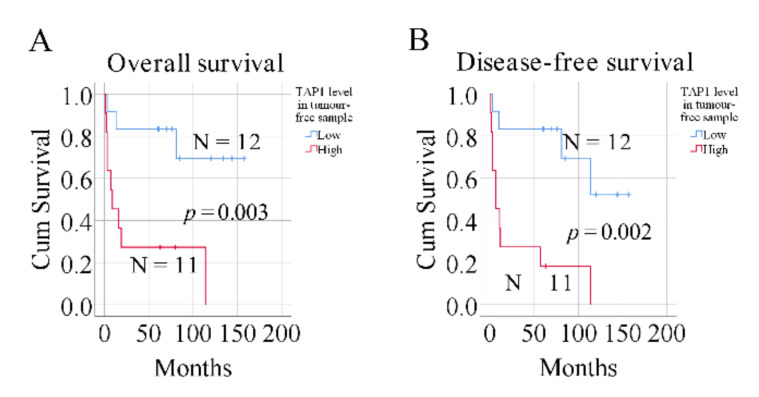
The influence of *TAP1* levels in tumor-free samples on patient survival. Kaplan–Meier curves of overall (**A**) and disease-free (**B**) survival. Blue lines represent patients with low *TAP1* levels, and red lines patients with high *TAP1* levels. Log-rank test for overall (*p* = 0.003) and disease-free survival (*p* = 0.002). N = number of samples.

Using univariate Cox regression survival analysis, we found that low expression of the *TAP1* gene in tumor-free samples was significantly associated with better patient survival (*p* < 0.05 for both overall and disease-free survival; Table 1). However, regarding TAP1 expression levels in tumor samples, no correlation to survival was found (Table 1). Previously, using the same samples, we reported that cytolytic activity in the tumor-free tongue is a useful prognostic factor [6]. Therefore, in the subsequent multivariate Cox regression analysis, we considered cytolytic activity, age at diagnosis, sex, clinical stage, T stage, and N stage as covariates. The results showed that *TAP1* levels in tumor-free samples remained significant as an independent prognostic factor for overall (*p* = 0.031) and disease-free survival (*p* = 0.005) (Table 2). Finally, dividing patients into *TAP1*-high and *TAP1*-low groups according to the median *TAP1* level in tumor-free samples demonstrated that patients with lower *TAP1* mRNA levels in tumor-free samples had better overall (*p* = 0.003, Figure 2A) and disease-free survival (*p* = 0.002, Figure 2B). As shown in Table 3, Fisher’s exact test indicated that there was no significant correlation between TAP1 levels and different clinical factors (*p* > 0.05).

**Figure 3:**
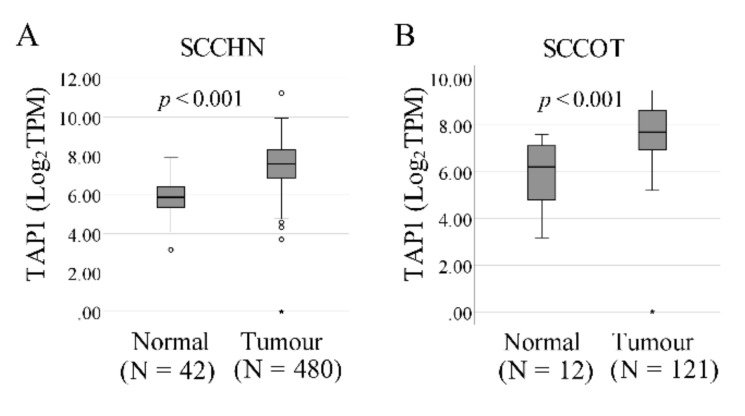
TCGA-HNSC (The Cancer Genome Atlas-head and neck squamous cell carcinoma) cohort data. Plots show *TAP1* levels in tumors and tumor-free samples, the latter denominated “normal” in TCGA, for (**A**) the whole group of squamous cell carcinoma of the head and neck–SCCHN (*p* < 0.001) and (**B**) for squamous cell carcinoma of the tongue (SCCOT) only (*p* < 0.001).

**Figure 4:**
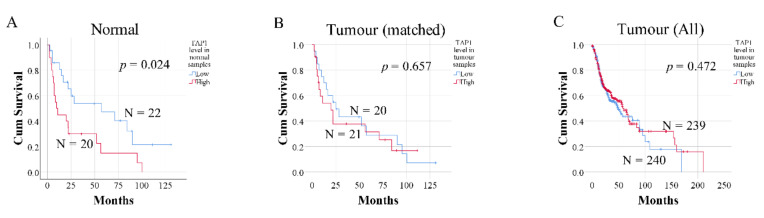
The influence of *TAP1* levels in tumor-free samples from the TCGA-HNSC cohort on patient survival. Kaplan–Meier curves of overall survival for (**A**) tumor-free samples (denominated “normal” by TCGA), (**B**) matched tumor samples, and (**C**) all SCCHN tumors available in the TCGA database. Blue lines represent patients with low *TAP1* levels, and red lines indicate patients with high *TAP1* levels. N = number of samples

To independently evaluate the prognostic value of *TAP1*, we downloaded gene expression and clinical data from The Cancer Genome Atlas, TCGA,-head and neck squamous cell carcinoma (HNSC) cohort. In this database, *TAP1* levels (achieved from RNA sequencing) were available for 480 tumors and 42 tumor-free samples, showing significantly higher *TAP1* levels in tumors (*p* < 0.001, Figure 3A). *TAP1* levels were also higher in SCCOT (N = 121) compared to tumor-free controls (N = 12, *p* < 0.001, Figure 3B). However, samples from different subsites of the head and neck were included in the external dataset as tumor-free controls, rather than tumor-free tongue contralateral to the SCCOT. Thus, no relevant comparison between *TAP1* in tumor-free samples of SCCOT and the clinical outcome was possible using TCGA patient samples. Therefore, we used the median *TAP1* mRNA level to divide the mixed group of 42 tumor-free samples into *TAP1*-low and *TAP1*-high groups. There was no significant association between *TAP1* levels and different clinical factors (Fisher’s exact test, *p* > 0.05, Table 4). Kaplan–Meier survival analysis showed that low *TAP1* in tumor-free tissues was significantly associated with better overall survival (log-rank test, *p*-value = 0.024, Figure 4A). Cox regression also showed that *TAP1*-low patients had better overall survival than *TAP1*-high patients (*p* = 0.028), also when considering cytolytic activity, age at diagnosis, sex, stage, and T and N values as covariates (*p* = 0.011, Table 5). Using Kaplan–Meier analysis on *TAP1* levels in 41 out of 42 matched SCCOT samples with follow up data available, no difference in overall survival was found between *TAP1*-low and *TAP1*-high patients (Figure 4B). Nor was there a difference in overall survival between *TAP1*-low and *TAP1*-high patients when considering the 479 out of 480 tumor samples with follow-up data available from the TCGA dataset (Figure 4C).

## 3. Discussion

Early detection of SCCOT improves its prognosis as well as diminishes the need for extensive surgical treatment. In the search for valuable prognostic factors, the clinically normal tissue adjacent to a tumor that remains in the patient after treatment has attracted attention. Field cancerization is a well-known phenomenon for tumors within the head and neck area [4] and we recently showed that increased cytolytic activity in clinically normal tongue contralateral to an SCCOT correlates to better survival [6]. Cytolytic activity is measured as levels of factors (perforin and granzymes) released by cytotoxic CD8^+^ T cells and NK cells, and high cytolytic activity is thus indicative of an immunologically active tissue. 

The heterodimer TAP is an important factor in formation of the MHC I antigen, acting as a complex and important factor in CD8^+^ T cell immune surveillance [8]. The TAP proteins, TAP1 and TAP2, are essential for antigen processing [8]. Whereas levels of *TAP2* were almost unchanged in the 21 tumor-free samples studied here, the group with *TAP1* mRNA levels below the median showed significantly better overall and disease-free survival when considering age, sex, cytolytic activity, and tumor stage. 

The results showed an inverse correlation between *TAP1* and the previous results on cytolytic activity in clinically tumor-free tongue contralateral to the SCCOT in relation to prognosis. Whereas increased cytolytic activity could be part of the inflammatory response in the tumor vicinity and thus play a protective role, the importance of decreased levels of *TAP1* is harder to explain as it is not well known how its expression is regulated under normal conditions. Whereas low levels of *TAP1* are an indicator of better survival, increased levels could serve as a sign of malignancy in tumor-free tissue contralateral to a treated SCCOT. This is, however, debatable due to the difficulty of defining “high” levels in tumor-free samples. Importantly, downregulation of *TAP1* in SCCHN has been reported previously [14,15,16]. A higher frequency of TAP down regulation has also been reported in metastatic compared to primary SCCHN [12]. By down regulating TAP causing loss of MHC class I antigen on the surface, tumors can escape cytotoxic T cell recognition [11]. Unexpectedly in our study, we found a gradual increase in TAP1 levels from healthy control to tumor-free to tumor samples. Conflicting results regarding the prognostic value of tumor TAP1 were also observed. When analyzing TAP1 in SCCOT, we did not find any correlation between expression levels and survival. In accordance with our mRNA data, TAP1 protein did not shown any correlation to survival in SCCHN tumors [14]. However, in esophageal cancer, upregulation of TAP1 protein in cancer tissues was found to be an unfavorable prognostic factor [17]. Differences between studies in terms of cancer subtypes and research methods, such as different assays and various scoring strategies, may be responsible for some of the discrepancies. In our group of SCCOTs, it could thus be interesting to map the expression of the TAP1 protein and its potential clinical relevance in comparison to the RNA-data shown here.

Overexpression of the TAP1 protein has been suggested as an indicator of aggressiveness in breast cancers, which is thought to be due either to a lower capacity of more advanced tumors to downregulate TAP, or to a higher concentration of immune infiltrates in the microenvironment producing IFN-γ, which causes upregulation of the TAP subunits [18]. In colorectal cancer, decreased levels of TAP1 were seen in tumors with perineural invasion, making downregulation of TAP1 a prognostic factor for patients with stage I and II disease [19].

As TAP plays a key role in CD8^+^ T cell immune surveillance, it would be interesting to see if the levels of TAP influence the therapeutic outcome in SCCOT patients treated with immune checkpoint inhibitors such as PD-1 or PD-L1 antibodies. 

In summary, the present findings illustrate the importance of studying the clinically tumor-free tongue contralateral to SCCOT in the search for relevant field changes that could be of diagnostic and/or prognostic importance. This tissue, remaining in the patient after treatment, is of utmost importance as it is easily accessible for different kinds of analyses at regular follow up. Our results, which show that *TAP1* levels in tumor-free tongue contralateral to the SCCOT correlate with survival, are an important contribution to early diagnostics and follow up of SCCOT, improving our overall understanding of this complicated and severe disease. 

## 4. Materials and Methods

### 4.1. Patient Material and Ethical Approval

The study comprised a total of 31 patients (15 men and 16 women) with a median age of 64 years (range 19–87 years) treated surgically and/or with radiotherapy for SCCOT at the Department of Otorhinolaryngology and Head & Neck Surgery, Norrland’s University Hospital, Umeå, Sweden. Approval for the study was obtained by the local Ethical Committee at Umeå University (Dnr 08-003M). Written informed consent was obtained from each participant. All samples were collected before treatment. For patients with status “alive disease-free”, the minimum follow up time was 5 years. 

Biopsies had been taken from the tumor in 29 of the patients and from the clinically tumor-free tongue on the contralateral side of the tumor in 21 of these. From the remaining two patients with SCCOT, only tumor-free samples were available. Tongue biopsies from 14 healthy individuals served as the control group. Clinical information for patients and controls is shown in Table 6. 

### 4.2. RNA Isolation and Gene Expression Profiling

As previously reported, RNA isolation and gene expression profiling were performed on 29 tumors, 23 tumor-free samples, and 14 healthy controls [6,13]. Briefly, biopsies were fresh-frozen in liquid nitrogen for further storage until RNA extraction. A total of 200 ng RNA was used for gene expression profiling with Illumina HumanHT-12 v4 Expression BeadChip (Illumina Inc., San Digo, CA, USA). Raw data are available at ArrayExpress under accession numbers E-MTAB-4678 and E-MTAB-5534. 

### 4.3. Prognostic Factor Analysis

Cox’s regression model was performed for survival analysis. Multivariate Cox regression analysis was also performed, with cytolytic activity, sex, age at diagnosis, and TNM stage as covariates. Patients were divided into high or low expression groups according to the median gene expression value in the tumor-free samples. Kaplan–Meier with log-rank tests were used to compare survival curves between groups. Fisher’s exact test was used to investigate the correlation between gene expression and clinical factors. All tests were conducted in IBM SPSS Statistics 26 (IBM Corp., Armonk, NY, USA). A two-sided *p*-value < 0.05 was considered significant.

### 4.4. Confirmation of Microarray Data Using RT-qPCR

Microarray data were confirmed using reverse transcription quantitative PCR (RT-qPCR) of tongue tissue from 10 of the healthy controls and 8 of the paired tumor/tumor-free samples from patients with SCCOT, using the QuantStudio 6 Flex real-time PCR system (Thermo Fisher Scientific, Waltham, MA, USA). Custom primers used were *TAP1* (forward: CTGGACTCCCTCAGGGCTAT, reverse: GGTTTCCGGATCAATGCTCG). As reference genes, *RPL13A* (forward: CACGAGGTTGGCTGGAAGTA, reverse: ACGTTCTTCTCGGCCTGTTT) and *GAPDH* (forward: GCCCTCAACGACCACTTTGT, reverse: TTACTCCTTGGAGGCCATGTG) were used. All primers were obtained from Thermo Fisher Scientific. Gene expression was quantified using the relative standard curve method. Spearman correlation coefficient (rho) was calculated to evaluate correlation strength between microarray and RT-qPCR results.

### 4.5. The Cancer Genome Atlas (TCGA) Data Collection and Analysis

Gene expression data from the TCGA head and neck cancer cohort was downloaded using the International Cancer Genome Consortium (ICGC) data portal (https://dcc.icgc.org/). Clinical data were downloaded from TCGA’s data portal (https://portal.gdc.cancer.gov). A prognostic factor analysis, as above, was performed to study the impact of gene expression on clinical outcome. 

## Figures and Tables

**Table 1:** Univariate Cox regression analysis of *TAP1* and *TAP2*.

Sample	Gene	*p*-Value (Cox-Regression Overall Survival Analysis)	*p*-value (Cox-Regression Disease-Free Survival Analysis)
Tumor-free	*TAP1*	0.042	0.026
	*TAP2*	0.828	0.987
Tumor	*TAP1*	0.772	0.892
	*TAP2*	0.158	0.315

**Table 2:** Univariate and multivariate Cox regression analysis of risk factors for survival.

		Univariate				Multivariate			
		*p*-value	Hazard Ratio	95% Confidence Interval		*p*-Value	Hazard Ratio	95% Confidence Interval	
				Lower	Upper			Lower	Upper
Overall survival	TAP1	0.042	1.836	1.023	3.294	0.031	12.650	1.267	126.283
	Cytolytic activity	0.082	2.931	0.871	9.861	0.176	3.271	0.588	18.212
	Age	0.607	1.010	0.972	1.049	0.799	1.007	0.954	1.063
	Sex	0.124	2.531	0.775	8.271	0.217	2.877	0.538	15.394
	Stage	0.013	1.952	1.149	3.318	0.064	0.046	0.002	1.201
	T	0.012	1.912	1.152	3.172	0.034	33.982	1.308	882.785
	N	0.011	2.482	1.233	4.996	0.364	1.819	0.500	6.622
Disease-free survival	TAP1	0.029	1.844	1.066	3.189	0.005	19.886	2.480	159.446
	Cytolytic activity	0.101	2.657	0.826	8.549	0.092	4.903	0.773	31.087
	Age	0.415	1.016	0.978	1.055	0.414	1.023	0.968	1.081
	Sex	0.099	2.509	0.841	7.486	0.236	2.399	0.565	10.188
	Stage	0.033	1.659	1.042	2.641	0.013	0.024	0.001	0.460
	T	0.028	1.674	1.057	2.650	0.010	52.561	2.581	1070.378
	N	0.016	2.285	1.163	4.490	0.176	2.373	0.679	8.285

**Table 3:** Clinical variables and their associations with *TAP1*.

		*TAP1* Level		*p*-Value
		Low	High	
Age (years)	≤65	7	4	0.414
	>65	5	7	
Sex	Female	4	7	0.220
	Male	8	4	
Stage	I, II	9	6	0.400
	III, IV	3	5	
T	T1, T2	10	7	0.371
	T3, T4	2	4	
N	N = 0	10	8	0.640
	N > 0	2	3	

**Table 4:** Clinical variables and their associations with TAP1 (TCGA data).

		*TAP1* Level		*p*-Value
		Low	High	
Age (years)	≤65	15	10	0.346
	>65	7	10	
Sex	Female	5	8	0.320
	Male	17	12	
Stage	I, II	17	17	0.700
	III, IV	5	3	
T	T1, T2	9	9	1.000
	T3, T4	13	11	
N	N = 0	16	15	1.000
	N > 0	5	5	

**Table 5:** Survival analysis with Cox regression on tumor-free samples in the TCGA HNSC cohort.

	Univariate				Multivariate			
	*p*-value	Hazard Ratio	95% Confidence Interval		*p*-Value	Hazard Ratio	95% Confidence Interval	
			Lower	Upper			Lower	Upper
***TAP1*** (high vs. low)	0.028	2.253	1.093	4.644	0.011	4.405	1.401	13.847
Cytolytic activity	0.447	1.318	0.647	2.683	0.217	0.476	0.146	1.547
Age	0.311	1.017	0.984	1.051	0.366	1.018	0.979	1.060
Sex	0.554	0.786	0.355	1.743	0.994	1.003	0.421	2.388
Clinical stage	0.125	1.340	0.922	1.950	0.632	1.275	0.472	3.445
**T stage**	0.194	1.310	0.871	1.971	0.801	1.105	0.509	2.397
**N stage**	0.074	1.711	0.949	3.086	0.408	1.441	0.606	3.426

**Table 6:** Clinicopathological data on SCCOT patients and healthy controls.

No	ID	Sample^¤^	Age	Sex	TNM	Stage	Localization ^#^	Status	Follow-up Months	Months to Recurrence
1	p40	1	80	Female	T4N2bM0	IV	3	DWD	1	
2	p42	1	68	Female	T2N0M0	II	1	DWD	9	7
3	p14	2	77	Female	T2N1M0	III	2	DDF	189	
4	p24	2	64	Male	T1N0M0	I	1	ADF	195	
5	p29	2	64	Female	T2N0M0	II	2	DWD	29	20
6	p68	2	62	Male	T2N0M0	II	1	DOD	9	6
7	p70	2	71	Male	T1N0M0	I	2	ADF	134	
8	p82	2	19	Female	T4N0M0	IV	2	DOD	18	12
9	p83	2	64	Female	T1N0M0	I	2	ADF	119	
10	p92	2	63	Female	T2N0M0	II	2	DOD	20	6
11	p11	3	78	Male	T2N0M0	II	2	DWD	3	
12	p35	3	24	Female	T2N0M0	II	1	DOD	13	10
13	p49	3	52	Female	T4N2cM0	IV	3	DWD	3	
14	p51	3	74	Male	T2N0M0	II	1	ADF	157	
15	p56	3	40	Female	T2N2bM0	IV	3	DOD	16	12
16	p58	3	61	Male	T1N0M0	I	1	ADF	144	
17	p59	3	68	Female	T2N0M0	II	1	DOD	7	
18	p61	3	69	Male	T4aN0M0	IV	3	DDF	81	
19	p65	3	81	Female	T2N0M0	II	3	ADF	134	114
20	p73	3	80	Male	T4aN0M0	IV	3	DOD	19	11
21	p76	3	58	Male	T4aN0M0	IV	3	DDF	114	
22	p79	3	60	Male	T1N0M0	I	2	ADF	120	
23	p85	3	87	Female	T2N0M0	II	1	DOD	2	2
24	p98	3	31	Male	T2N0M0	II	3	ADF	85	
25	p105	3	63	Male	T1N0M0	I	2	ADF	80	57
26	p111	3	31	Female	T1N0M0	I	2	ADF	76	
27	p119	3	66	Male	T2N0M0	II	2	ADF	70	
28	p124	3	54	Male	T4aN2bM0	IV	3	DOD	3	
29	p131	3	74	Female	T2N0M0	II	2	ADF	63	
30	p137	3	71	Female	T2N0M0	II	2	ADF	61	
31	p138	3	50	Male	T2N1M0	III	2	ADF	60	
32	NT1	4	32	Female						
33	NT2	4	49	Female						
34	NT3	4	25	Female						
35	NT4	4	30	Male						
36	NT5	4	27	Male						
37	NT6	4	42	Female						
38	NT7	4	32	Female						
39	NT8	4	41	Female						
40	NT9	4	35	Female						
41	NT10	4	57	Male						
42	NT11	4	45	Male						
43	NT12	4	37	Male						
44	NT13	4	48	Female						
45	NT14	4	59	Female						

Note: 1, only tumor-free sample; 2, only tumor sample; 3, tumor-free and tumor samples were collected, 4, healthy controls; # 1, tongue; 2, lateral border of the tongue; 3, tongue with overgrowth outside the mobile tongue; Status: DWD, dead with disease; DDF, dead disease-free; ADF, alive disease-free; DOD, dead of disease.
